# Applying Different Independent Component Analysis Algorithms and Support Vector Regression for IT Chain Store Sales Forecasting

**DOI:** 10.1155/2014/438132

**Published:** 2014-06-05

**Authors:** Wensheng Dai, Jui-Yu Wu, Chi-Jie Lu

**Affiliations:** ^1^International Monetary Institute, Financial School, Renmin University of China, Beijing 100872, China; ^2^Department of Business Administration, Lunghwa University of Science and Technology, Taoyuan County 33306, Taiwan; ^3^Department of Industrial Management, Chien Hsin University of Science and Technology, Taoyuan County 32097, Taiwan

## Abstract

Sales forecasting is one of the most important issues in managing information technology (IT) chain store sales since an IT chain store has many branches. Integrating feature extraction method and prediction tool, such as support vector regression (SVR), is a useful method for constructing an effective sales forecasting scheme. Independent component analysis (ICA) is a novel feature extraction technique and has been widely applied to deal with various forecasting problems. But, up to now, only the basic ICA method (i.e., temporal ICA model) was applied to sale forecasting problem. In this paper, we utilize three different ICA methods including spatial ICA (sICA), temporal ICA (tICA), and spatiotemporal ICA (stICA) to extract features from the sales data and compare their performance in sales forecasting of IT chain store. Experimental results from a real sales data show that the sales forecasting scheme by integrating stICA and SVR outperforms the comparison models in terms of forecasting error. The stICA is a promising tool for extracting effective features from branch sales data and the extracted features can improve the prediction performance of SVR for sales forecasting.

## 1. Introduction

Independent component analysis (ICA) is one of the most widely applied blind source separation (BSS) techniques for separating the source from the received signals without any prior knowledge of the source signal [1]. The goal of ICA is to recover independent sources when given only sensor observations that are unknown mixtures of the unobserved independent source signals. It has been investigated extensively in image processing, time series forecasting, and statistical process control [1–6]. For example, Oja et al. [2] applied linear ICA to foreign exchange rate time series prediction. They first used linear ICA to estimate the independent components and mixing matrix from the observed time series dataset and then filtered the independent components (to reduce the effects of noise) through linear and nonlinear smoothing techniques. Finally, autoregression (AR) modeling was employed to predict the smoothed independent components. Cao and Chong [3] employed ICA as a feature extraction tool in developing a support vector machine (SVM) forecaster. The independent components (ICs) were considered features of the forecasting data and used to build the SVM forecasting model.

Lu et al. [4] proposed a hybrid scheme which integrates ICA, engineering process control (EPC), and backpropagation neural network (BPN) to recognize shift and trend patterns in correlated processes. Lu [5] developed an ICA-based disturbance separation scheme to diagnose shift patterns with different levels of shift magnitudes in a correlated process. Lu et al. [6] proposed a two-stage forecasting model by integrating linear ICA and support vector regression (SVR) for financial time series. They first applied linear ICA to the forecasting variables to generate the independent components. After identifying and removing the ICs containing the noise, the rest of the ICs were then used to reconstruct the forecasting variables which contained less noise and served as the input variables of the SVR forecasting model.

For time series forecasting problems, the first important step is usually to use feature extraction to reveal the underlying/interesting information that cannot be found directly from the observed data. The performance of predictors can be improved by using the features as inputs [6–10]. Therefore, the two-stage forecasting scheme by integrating feature extraction method and prediction tool is a well-known method in literature [7–9]. The basic ICA is usually used as a novel feature extraction technique to find independent sources (i.e., features) for time series forecasting [1–3, 6–8]. The independent sources called independent components (ICs) can be used to represent hidden information of the observable data. The basic ICA has been widely applied in different time series forecasting problems, such as stock price prediction and exchange rate forecasting [2, 6, 11, 12]. However, there are only very few articles utilizing ICA in sales forecasting. Lu and Wang [13] combined ICA, growing hierarchical self-organizing maps (GHSOM), and SVR to develop a clustering-based sales forecasting model for predicting sales of computer dealer.

The basic ICA was originally developed to deal with the problems similar to the “cocktail party” problem in which many people are speaking at once. It assumed that the extracted ICs are independent in time (independence of the voices) [14]. Thus, the basic ICA is also called temporal ICA (tICA). However, for some application data such as biological time series and functional magnetic resonance imaging (fMRI) data, it is more realistic assumed that the ICs are independent in space (independent of the images or voxel) [15, 16]. This ICA model is called spatial ICA (sICA). Besides, spatiotemporal ICA (stICA) based on the assumption that there exist small dependences between different spatial source data and between different temporal source data is also proposed [15, 16]. In other words, stICA maximizes the degree of independence over space as well as over time, without necessarily producing independence in either space or time [15, 16]. In short, there are three different ICA algorithms. tICA seeks a set of ICs which are strictly independent in time. On the contrary, sICA tries to find a set of ICs which are strictly independent in space. stICA seeks a set of ICs which are not strictly independent over time nor space.

Many studies have been reported on using sICA and/or stICA algorithms to extract the distinguishability information from time series data. Calhoun et al. [14] used sICA and tICA algorithms to extract features from fMRI data. They found that sICA and tICA can have diverging results, depending upon the characteristics of the underlying signals to be estimated. But, they also indicated that sICA and tICA algorithms can produce similar results when the signals are uncorrelated in both the spatial and the temporal dimensions. Stone et al. [16] applied stICA for event-related fMRI data. Their results showed that the performance of stICA was superior to those of principal component analysis (PCA), sICA and tICA. Kim et al. [17] employed sICA, tICA, and stICA for clustering genes and finding biologically meaningful modes. The results showed that tICA was more useful than sICA and stICA in the task of gene clustering and that the modes found by stICA were better than that of sICA and tICA. Castells et al. [18] used sICA and stICA algorithms for analyzing simulated and real electrocardiograms (ECGs) data and found the stICA algorithm outperformed the sICA model.

Sales forecasting is one of the most important issues for information technology (IT) companies [13, 19, 20] as IT companies face a competitive environment, with rapid changes to product specifications, intense competition, and rapidly eroding prices. By predicting consumer demand before selling, sales forecasting helps to determine the appropriate number of products to keep in inventory, thereby preventing over- or understocking. Moreover, since an IT chain store has many branches, how to construct an effective sales forecasting model is a challenging task for managing the IT chain store sales.

The sales of a branch of an IT chain store may be affected by other neighboring branches of the same IT chain store. Therefore, to forecast sales of a branch, the historical sales data of this branch and its neighboring branches will be good predictor variables. The historical sales data of the branches of an IT chain store are highly correlated in space or time or both. Thus, three different ICA algorithms are used in this study to extract features from the branch sales data of an IT chain store. The feature extraction performance of the three different ICA algorithms is compared by using the two-stage forecasting scheme.

In this study, we propose a sales forecasting model for the branches of an IT chain store by integrating ICA algorithms and SVR. SVR based on statistical learning theory is an effective neural network algorithm and has been receiving increasing attention for solving nonlinear regression estimation problems. The SVR is derived from the structural risk minimization principle to estimate a function by minimizing an upper bound of the generalization error [21]. Due to the advantages of the generalization capability in obtaining a unique solution, SVR can lead to great potential and superior performance in practical applications. It has been successfully applied in time series forecasting problem, such as sales data [13, 19, 20], traffic flow [22–24], electric load [25–27], wind speed [28], and financial time series data [6, 7, 10, 29, 30].

In the proposed sales forecasting scheme, we first use three different ICA algorithms (i.e., tICA, sICA, and stICA) on the predictor variables to estimate ICs. The ICs can be used to represent underlying/hidden information of the predictor variables. The ICs are then used as the input variables of the SVR for building the prediction model. In order to evaluate the performance of the three different ICA algorithms, a real branch sales data of an IT chain store is used as the illustrative example.

The rest of this paper is organized as follows.  Section 2 gives brief overviews of temporal ICA, spatial ICA, and spatiotemporal ICA and SVR. The sales forecasting scheme is described in Section 3.  Section 4 presents the experimental results and this paper is concluded in Section 5.

## 2. Methodology

### 2.1. Temporal, Spatial, and Spatiotemporal ICA

In general, stICA finds a linear decomposition, by maximizing the degree of independence over space as well as over time, without necessarily producing independence in either space or time. It permits a tradeoff between the independence of arrays and the independence of time courses. Different from stICA, tICA enforces independence constraints over time, to seek a set of independent time courses. While, sICA compels independence constraints over space, to find a set of independent arrays [17].

Let **X** = [**x**_1_, **x**_2_,…,**x**_*m*_]^*T*^  be an input matrix of size *m* × *n*,   *m* ≤ *n*, consisting of observed mixture signals *x*_*i*_ of size 1 × *n*,   *i* = 1, 2,…, *m*. Suppose that the singular value decomposition (SVD) of **X** is given by **X** = **U** **D** **V** ^*T*^, where **U** ∈ *R* ^*m*×*k*^, *k* ≤ *m* corresponds to eigenarrays, **V** ∈ *R* ^*n*×*k*^ is associated with eigensequences, and **D** is a diagonal matrix containing singular values. Following the notations in Stone [15], it is defined $\tilde{X}=UDV^{T}=\tilde{U}{\tilde{V}}^{T}$, where $\tilde{U}={UD}^{1/2}$ and $\tilde{V}={VD}^{1/2}$.

For temporal ICA (tICA), it embodies the assumption that $\tilde{V}$ can be decomposed: $\tilde{V}=PA_{P}$, where **A**_*P*_ is an *k* × *k* mixing matrix and **P** is an *n* × *k* matrix of *k* statistically independent temporal signals. tICA can be used to obtain the decomposition $p_{T}=\tilde{V}W_{P}$. **W**_*P*_  is a permuted version of **A**_*P*_^−1^. The vector **p**_*T*_ is a set of extracted temporal signals and is a scale version of exactly one column vector in matrix **P**. This is achieved by maximizing the entropy *h*_*T*_ = *H*(**Y**) of **Y** = *τ*(**P**_*T*_), where *τ* is approximates the cdf of the temporal source signals.

For spatial ICA (sICA), it is assumed that $\tilde{U}$ can be decomposed as $\tilde{U}=SA_{S}$, where **A**_*S*_ is a *k* × *k* mixing matrix and **S** is an *m* × *k* matrix of *k* statistically independent spatial signals. sICA can be applied to generate the decomposition $y_{S}=\tilde{U}W_{S}$, where **W**_*S*_  is a permuted version of **A**_*S*_^−1^. The vector **y**_*S*_ is a scale version of exactly one column vector in matrix **S** and is a set of extracted spatial signals. This is achieved by maximizing the entropy *h*_*S*_ = *H*(**Z**) of **Z** = *δ*(**y**_*S*_), where *δ* is approximates the cdf of the spatial source signals.

In spatiotemporal ICA (stICA), it is trying to find the decomposition $\tilde{X}=S\Lambda P^{T}$, where **S** is an *m* × *k* matrix with a set of *k* statistically independent spatial signals, **P** is an *n* × *k* matrix of *k* mutually independent temporal signals, and Λ is a diagonal scaling matrix and is required to ensure that **S** and **P** have amplitudes appropriate to their respective cdfs *δ* and *τ*. Under the condition of $\tilde{X}=\tilde{U}{\tilde{V}}^{T}$, there exist two *k* × *k* un-mixing matrices **W**_*P*_ and **W**_*S*_, such that $P=\tilde{V}W_{P}$ and $S=\tilde{U}W_{S}$. Then, if **W**_*S*_Λ**W**_*T*_ = **I**, the following relation holds: $\tilde{X}=S\Lambda P^{T}=\tilde{U}W_{S}\Lambda ({\tilde{V}W_{P})}^{T}=\tilde{U}{\tilde{V}}^{T}$. We can estimate the **W**_*P*_ and **W**_*S*_ by maximizing an objective function associated with spatial and temporal entropies at the same time. That is, the objective function for stICA has the following form: *h*_*ST*_(**W**_*S*_, Λ) = *αH*(**Z**) + (1 − *α*)*H*(**Y**), where *α* (0.5 is used in this study) defines the relative weighting for spatial entropy and temporal entropy. More details on tICA, sICA, and stICA can be found in [14–16].

### 2.2. Support Vector Regression

Support vector regression (SVR) is an artificial intelligent forecasting tool based on statistical learning theory and structural risk minimization principle [21]. The SVR model can be expressed as the following equation [21]:

(1) $$\begin{array}{c} f\left(x\right)=\left(z\emptyset \left(x\right)\right)+b, \end{array}$$

where **z** is weight vector, *b* is bias, and *∅*(*x*) is a kernel function which uses a nonlinear function to transform the nonlinear input to be linear mode in a high dimension feature space.

Traditional regression gets the coefficients through minimizing the square error which can be considered as empirical risk based on loss function. Vapnik [21] introduced the so-called *ε*-insensitivity loss function to SVR. Considering empirical risk and structure risk synchronously, the SVR model can be constructed to minimize the following programming:

(2) Min⁡:(zTz)2+C∑i(φi+φi∗)Subject  to:yi−zTxi−b≤ε+φizTxi+b−yi≤ε+φi∗φi,φi∗≥0,

where *i* = 1,…, *n* is the number of training data; (*φ*_*i*_ + *φ*_i_*) is the empirical risk; *ε* defined the region of *ε*-insensitivity, when the predicted value falls into the band area, the loss is zero. Contrarily, if the predicted value falls out the band area, the loss is equal to the difference between the predicted value and the margin; **z** ^*T*^ **z**/2 is the structure risk preventing overlearning and lack of applied universality; *C* is modifying coefficient representing the tradeoff between empirical risk and structure risk.

After selecting proper modifying coefficient (*C*), width of band area (*ε*), and kernel function (*∅*(*x*)), the optimum of each parameter can be resolved though Lagrange function. Cherkassky and Ma [31] proposed that radial basis function (RBF), defined as Φ(*x*_*i*_, *x*_*j*_) = exp⁡⁡⁡(−||*x*_*i*_−*x*_*j*_||^2^/2*σ* ^2^), is suited for solving most forecasting problems. So this paper uses RBF with parameter *σ* = 0.2 as kernel function in SVR modeling. The performance of SVR is mainly affected by the setting of parameters *C* and *ε* [21, 31]. There are no general rules for the choice of *C* and *ε*. This study uses exponentially growing sequences of *C* and *ε* to identify good parameters [32]. The parameter set of *C* and *ε* which generate the minimum forecasting mean square error (MSE) is considered as the best parameter set.

## 3. Proposed Sales Forecasting Scheme

**Figure 1:**
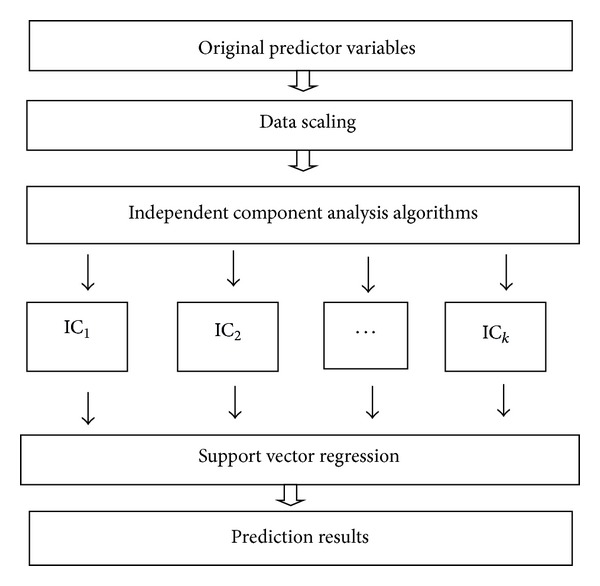
The proposed sales forecasting scheme.

This study uses a two-stage sales forecasting scheme. In this scheme, we use different ICA algorithms as feature extraction method and utilize support vector regression as prediction tool. The schematic representation of the proposed sales forecasting scheme is illustrated in Figure 1.

As shown in Figure 1, the first step of the proposed sales forecasting scheme is data scaling. In this step, the original datasets and prediction variables are scaled into the range of [−1.0,1.0] by utilizing min-max normalization method. The min-max normalization method converts a value *x* of variable *X* to *x*′ in the range [−1.0,1.0] by computing *x*′ = −1 + (2(*x* − min⁡⁡*X*)/(max⁡⁡*X* − min⁡⁡*X*)), where max⁡⁡*X* and min⁡⁡*X* are the maximum and minimum values for attribute/variable *X*.

Then, the three different ICA algorithms including tICA, sICA, and stICA are used in the scaled data to estimate ICs. In the third step, the ICs contained hidden information of the prediction variables are used as input variables to construct SVR sales forecasting model. Since this study uses three ICA algorithms to extract features, based on the two-stage scheme, four sales forecasting methods including tICA-SVR, sICA-SVR, t-stICA-SVR, and s-stICA-SVR are presented in this study. For the tICA-SVR, the tICA algorithm is used to generate temporal ICs (called t_ICs). The sICA algorithm is utilized to estimate spatial ICs (called s_ICs) for sICA-SVR method. As stICA algorithm generates two different sets of ICs which are used to represent the temporal ICs (called t-st_ICs) and spatial ICs (called s-st_ICs), respectively; the t-stICA-SVR forecasting model using t-st_ICs as inputs and s-stICA-SVR prediction scheme applying s-st_ICs as prediction variables are developed.

## 4. Experimental Results

### 4.1. Datasets and Performance Criteria

**Figure 2:**
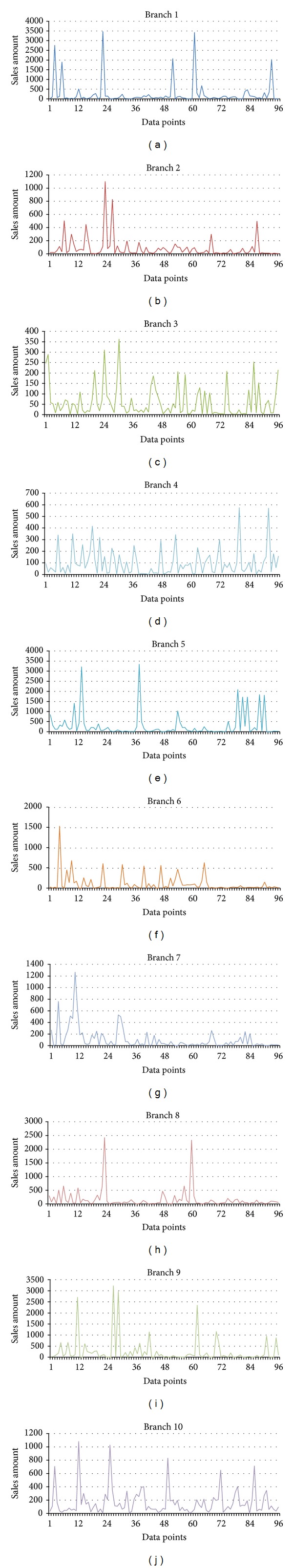
(a)–(j) The sales data of the 10 branches.

For evaluating the performance of the three different ICA algorithms for sales forecasting for IT chain store, a real weekly branch sales dataset of an IT chain store is used in this study. This data contains 10 neighboring branches. There are totally 96 data points in each branch. The first 70 data points (72.9% of the total sample points) are used as the training sample and the remaining 26 data points (27.1% of the total sample points) are used as testing sample. Figures 2(a)–2(j) show the sales data of the 10 branches, respectively. From Figures 2(a)–2(j), it can be seen that sales characteristics between the 10 branches are different. As these 10 branches are neighboring branches, if we want to forecast sales of one of the 10 branches (can be called target branch), the sales data of the rest 9 branches can be used as predictor variables. Therefore, the previous week's sales volume (T-1) of the target branch and the 9 neighboring branches are used as 10 predictor variables. The input matrices **X**_**tr**_ of size 10  ×  70 and **X**_**te**_ of size 10  ×  26 are then generated for training stage andtesting stage, respectively.

The prediction results of the four two-stage sales forecasting schemes including tICA-SVR, sICA-SVR, t-stICA-SVR, and s-stICA-SVR methods are compared to the SVR model without using ICA for feature extraction (called the single SVR model). All of the five forecasting schemes are used for one-step ahead forecasting of monthly sales data (i.e., one-month ahead forecasting). In building the SVR forecasting model, the LIBSVM package proposed by Chang and Lin [33] is adapted in this study.

The prediction performance is evaluated using the following statistical metrics, namely, the root mean square error (RMSE), mean absolute difference (MAD), and mean absolute percentage error (MAPE). RMSE, MAD, and MAPE are measures of the deviation between actual and predicted values. The smaller the values of RMSE, MAD, and MAPE, the closer are the predicted time series values to that of the actual value. The definitions of these criteria are as below:

(3) $$\begin{array}{c} \text{RMSE}=\sqrt{\frac{\sum_{i=1}^{n}{\left(T_{i}-P_{i}\right)}^{2}}{n}},\\ \text{MAD}=\frac{\sum_{i=1}^{n}{\left|T_{i}-P_{i}\right|}^{2}}{n},\\ \text{MAPE}=\frac{\sum_{i=1}^{n}\left|\left(T_{i}-P_{i}\right)/T_{i}\right|}{n}, \end{array}$$

where *T*_*i*_ and *P*_*i*_ represent the actual and predicted value at week *i*, respectively; *n* is the total number of data points.

### 4.2. Forecasting Results

In this study, 10 branches' sales data are used to assess the performance of the five forecasting methods. In this section, first, we use the sales data of Branch 1 as evaluation sample. That is, Branch 1 is the first target branch.

In the modeling of single SVR model for Branch 1, the scaled values of the 10 predictor variables are directly used as inputs. In selecting the parameters for modeling SVR, the parameter set (*C* = 2^11^, *ε* = 2^−7^) is used as the start point of grid search for searching the best parameters. The testing results of the SVR model with combinations of different parameter sets are summarized in Table 1. From Table 1, it can be found that the parameter set (*C* = 2^11^, *ε* = 2^−7^) gives the best forecasting result (minimum testing MSE) and is the best parameter set for single SVR model.

For the tICA-SVR model, first, the original predictor variables are scaled and then passed to tICA algorithm to estimate ICs, that is, features. The ICs are then used for building SVR forecasting model. Ten ICs are estimated by the tICA algorithm since 10 predictors are used. As the same process with above single SVR, the parameter set (*C* = 2^9^, *ε* = 2^−7^) is used as the start point of grid search.  Table 2 summarizes the testing results of the tICA-SVR model with combinations of different parameter sets. As Table 2 shows, the parameter set (*C* = 2^11^, *ε* = 2^−5^) gives the best forecasting result and is the best parameter set for the tICA-SVR model.

Using the similar process, the sICA-SVR model uses sICA algorithm to generate spatial ICs (i.e., s_ICs); the t-stICA-SVR model and s-stICA-SVR model utilize stICA algorithm to respectively estimate temporal ICs (i.e., t-st_ICs) and spatial ICs (i.e., s-st_ICs). Tables 3, 4, and 5 show the model selection results of the sICA-SVR, t-stICA-SVR, and s-stICA-SVR models, respectively. From the tables, it can be found that the best parameter sets for sICA-SVR, t-stICA-SVR, and s-stICA-SVR models are (*C* = 2^9^, *ε* = 2^−9^), (*C* = 2^11^, *ε* = 2^−7^), and (*C* = 2^11^, *ε* = 2^−9^), respectively.

The forecasting results of Branch 1 using the tICA-SVR, sICA-SVR, t-stICA-SVR, s-stICA-SVR, and single SVR models are computed and listed in Table 6. Table 6 depicts that the RMSE, MAD, and MAPE of the t-stICA-SVR model are, respectively, 20.369, 8.140, and 6.64%. It can be observed that these values are smaller than those of the tICA-SVR, sICA-SVR, s-stICA-SVR, and single SVR models. Therefore, the t-stICA-SVR model can generate the best prediction result for forecasting sales of Branch 1.

Using a similar modeling process as abovementioned, the five forecasting models are conducted for forecasting sales of Branch 2 to Branch 10.  Table 7 summarizes the forecasting results of Branch 2 to Branch 10 using the tICA-SVR, sICA-SVR, t-stICA-SVR, s-stICA-SVR, and single SVR models, respectively. Note that the values in Table 7 are MAPE values. It can be observed from the Table 7 that the t-stICA-SVR forecasting scheme has the smallest RMSE, MAD, and MAPE in comparison with the four comparison models in every branches. Thus, the t-stICA-SVR model can provide better forecasting precision and outperforms the four comparison methods in forecasting sales of IT chain store.

Moreover, it also can be seen from the Tables 6 and 7 that s-stICA-SVR outperforms the tICA-SVR, sICA-SVR, and single SVR models. Since the t-stICA-SVR and s-stICA-SVR can provide better forecasting results than the tICA-SVR and sICA-SVR models, it indicates that stICA algorithm can estimate more effective ICs and improve sales forecasting performance for IT chain store. Besides, from the Tables 6 and 7, we find that temporal ICs are more suitable for forecasting branch sales since the forecasting performance of t-stICA-SVR and tICA-SVR models is better than that of s-stICA-SVR and sICA-SVR models, respectively.

In order to further evaluate and compare the performance of the five forecasting schemes (i.e., tICA-SVR, sICA-SVR, t-stICA-SVR, s-stICA-SVR, and single SVR models), 3-month ahead and 6-month ahead forecasts are also considered in this study. The forecasting errors of the five abovementioned forecasting schemes under three different forecast horizons are computed and listed in Table 8. From Table 8, it can be found that the MAPE of the t-stICA-SVR scheme is all smaller than those of the tICA-SVR, sICA-SVR, s-stICA-SVR, and single SVR models for the three forecast horizons. It indicates that the t-stICA-SVR scheme can consistently yield a smaller deviation between the actual and predicated values and then effectively provide good forecasting accuracy in different forecast horizons. In addition, it also can be observed from Table 8 that the t-stICA-SVR and s-stICA-SVR schemes outperform the tICA-SVR, sICA-SVR, and single SVR models at all three forecast horizons. Based on the findings in Table 8, it reveals that stICA is a promising tool for extracting effective features from branch sales data and the extracted features can improve the prediction performance of SVR for sales forecasting. The experimental results are consistent with the conclusions of Stone et al. [16] and Kim et al. [17]. The stICA solution extracts the original source signal to a much greater extent than the tICA and sICA solution if the latent variables contain spatial and temporal information.

## 5. Conclusion

Forecasting sales of branches is a crucial aspect of the marketing and inventory management in IT chain store. In this paper, we used three different ICA algorithms including tICA, sICA, and stICA for sales forecasting and compared the feature extraction performance of the three different ICA algorithms. Four sales forecasting methods including tICA-SVR, sICA-SVR, t-stICA-SVR, and s-stICA-SVR were presented in this study. In the proposed sales forecasting methods, we first used three different ICA algorithms (i.e., tICA, sICA, and stICA) on the predictor variables to estimate ICs. The ICs can be used to represent underlying/hidden information of the predictor variables. The ICs are then used as the input variables of the SVR for building the prediction model. A real weekly sales data including 10 branches of an IT chain store was used for evaluating the performance of the sales forecasting methods. Experimental results showed that the t-stICA-SVR and s-stICA-SVR models can produce the lowest prediction error in forecasting sales of the 10 branches. They outperformed the comparison methods used in this study. Thus, compared to tICA and sICA algorithms, stICA algorithm can estimate more effective ICs and improve sales forecasting performance for IT chain store. Moreover, we also found that, compared to spatial ICs, the temporal ICs are more suitable features for forecasting branch sales.

## Figures and Tables

**Table 1:** Model selection results of the single SVR model.

*C*	*ε*	Training MSE	Testing MSE
2^9^	2^−5^	0.0521	0.0688
	2^−7^	0.0537	0.0678
	2^−9^	0.0552	0.0667
	2^−11^	0.0547	0.0664

2^11^	2^−5^	0.0712	0.0582
	2^−7^	**0.0399**	**0.0492**
	2^−9^	0.0407	0.0523
	2^−11^	0.0407	0.0567

2^13^	2^−5^	0.0539	0.0673
	2^−7^	0.0555	0.0655
	2^−9^	0.0561	0.0676
	2^−11^	0.0572	0.0672

**Table 2:** Model selection results of the tICA-SVR model.

*C*	*ε*	Training MSE	Testing MSE
2^7^	2^−3^	0.0401	0.0541
	2^−5^	0.0391	0.0523
	2^−7^	0.0380	0.0515
	2^−9^	0.0377	0.0495

2^9^	2^−3^	0.0396	0.0537
	2^−5^	0.0388	0.0529
	2^−7^	0.0379	0.0541
	2^−9^	0.0379	0.0592

2^11^	2^−3^	0.0390	0.0609
	2^−5^	**0.0374**	**0.0455**
	2^−7^	0.0376	0.0753
	2^−9^	0.0395	0.0508

**Table 3:** Model selection results of the sICA-SVR model.

*C*	*ε*	Training MSE	Testing MSE
2^9^	2^−7^	0.0410	0.0639
	**2** ^−9^	**0.0390**	**0.0478 **
	2^−11^	0.0395	0.0791
	2^−13^	0.0415	0.0533

2^11^	2^−7^	0.0416	0.0564
	2^−9^	0.0407	0.0555
	2^−11^	0.0398	0.0568
	2^−13^	0.0398	0.0622

2^13^	2^−7^	0.0421	0.0568
	2^−9^	0.0411	0.0549
	2^−11^	0.0399	0.0541
	2^−13^	0.0396	0.0520

**Table 4:** Model selection results of the t-stICA-SVR model.

*C*	*ε*	Training MSE	Testing MSE
2^7^	2^−3^	0.0324	0.0438
	2^−5^	0.0316	0.0423
	2^−7^	0.0307	0.0417
	2^−9^	0.0305	0.0401

2^9^	2^−3^	0.0320	0.0434
	2^−5^	0.0314	0.0428
	2^−7^	0.0306	0.0438
	2^−9^	0.0306	0.0479

2^11^	2^−3^	0.0304	0.0610
	2^−5^	0.0315	0.0493
	2^−7^	**0.0303**	**0.0369**
	2^−9^	0.0320	0.0411

**Table 5:** Model selection results of the s-stICA-SVR model.

*C*	*ε*	Training MSE	Testing MSE
2^9^	2^−7^	0.0343	0.0459
	2^−9^	0.0334	0.0453
	2^−11^	0.0325	0.0463
	2^−13^	0.0322	0.0506

2^11^	2^−7^	0.0320	0.0463
	2^−9^	**0.0314 **	**0.0421 **
	2^−11^	0.0326	0.0440
	2^−13^	0.0324	0.0423

2^13^	2^−7^	0.0334	0.0521
	2^−9^	0.0320	0.0389
	2^−11^	0.0321	0.0644
	2^−13^	0.0338	0.0435

**Table 6:** Forecasting results of Branch 1 using the five forecasting models.

Models	RMSE	MAD	MAPE
tICA-SVR	70.596	17.551	13.97%
sICA-SVR	104.946	50.635	21.32%
t-stICA-SVR	**20.369 **	**8.140 **	**6.64%**
s-stICA-SVR	33.757	10.922	13.11%
Single SVR	115.529	55.741	24.84%

**Table 7:** Sales forecasting results of Branch 2 to Branch 10.

Models	Branches								
	B2	B3	B4	B5	B6	B7	B8	B9	B10
tICA-SVR	16.45%	13.92%	15.61%	17.75%	15.39%	17.49%	12.33%	12.40%	14.20%
sICA-SVR	17.37%	22.12%	17.44%	17.95%	16.88%	17.71%	21.26%	16.36%	24.92%
t-stICA-SVR	**6.03%**	**7.20%**	**7.34%**	**4.81%**	**7.28%**	**5.03%**	**4.37%**	**4.33%**	**11.79%**
s-stICA-SVR	12.26%	13.86%	15.11%	16.07%	9.34%	15.04%	9.60%	10.75%	13.50%
Single SVR	26.93%	25.87%	21.58%	25.04%	21.76%	22.33%	28.75%	21.90%	30.36%

*Note: The values in this table are MAPE.

**Table 8:** Forecasting accuracy comparison of the five forecasting schemes under three different forecast horizons.

Models	Branches									
	B1	B2	B3	B4	B5	B6	B7	B8	B9	B10
1-month ahead forecast										
tICA-SVR	13.97%	16.45%	13.92%	15.61%	17.75%	15.39%	17.49%	12.33%	12.40%	14.20%
sICA-SVR	21.32%	17.37%	22.12%	17.44%	17.95%	16.88%	17.71%	21.26%	16.36%	24.92%
t-stICA-SVR	**6.64%**	**6.03%**	**7.20%**	**7.34%**	**4.81%**	**7.28%**	**5.03%**	**4.37%**	**4.33%**	**11.79%**
s-stICA-SVR	13.11%	12.26%	13.86%	15.11%	16.07%	9.34%	15.04%	9.60%	10.75%	13.50%
Single SVR	24.84%	26.93%	25.87%	21.58%	25.04%	21.76%	22.33%	28.75%	21.90%	30.36%

3-month ahead forecast										
tICA-SVR	18.41%	18.36%	20.29%	24.70%	26.71%	25.02%	18.62%	17.00%	21.17%	16.45%
sICA-SVR	28.90%	18.96%	30.12%	25.61%	26.78%	26.56%	19.88%	27.76%	24.20%	30.01%
t-stICA-SVR	**15.44%**	**13.40%**	**17.15%**	**15.98%**	**7.56%**	**15.14%**	**12.83%**	**11.42%**	**12.33%**	**19.51%**
s-stICA-SVR	19.16%	19.98%	17.63%	18.08%	20.45%	16.61%	20.17%	12.54%	17.77%	15.13%
Single SVR	33.68%	35.13%	29.02%	27.51%	29.06%	27.69%	28.05%	31.84%	25.16%	32.32%

6-month ahead forecast										
tICA-SVR	33.08%	29.36%	33.71%	35.05%	35.45%	29.95%	31.24%	25.77%	31.94%	31.48%
sICA-SVR	36.88%	30.30%	35.54%	32.14%	29.39%	32.35%	36.88%	35.80%	30.20%	38.63%
t-stICA-SVR	**20.31%**	**24.34%**	**22.20%**	**19.87%**	**22.80%**	**19.65%**	**23.89%**	**18.69%**	**22.71%**	**26.42%**
s-stICA-SVR	26.84%	27.31%	26.19%	27.46%	34.52%	23.28%	34.83%	28.13%	26.93%	30.45%
Single SVR	37.10%	40.48%	44.76%	36.82%	42.79%	38.80%	35.86%	47.77%	38.06%	50.27%

*Note: The values in this table are MAPE.
